# Curative response to combined targeted-immunotherapy for post-hepatectomy lymph node metastasis in sarcomatoid hepatocellular carcinoma: case report and literature review

**DOI:** 10.3389/fonc.2025.1591419

**Published:** 2025-09-18

**Authors:** Pan Liu, Song Zhang, Xiao-Ming Xin, Min Jing, Lie-Dong Wen, Xin Xiang, Shun-Hai Liu

**Affiliations:** ^1^ Department of Hepatobiliary Surgery, The First People’s Hospital of Neijiang, Neijiang, Sichuan, China; ^2^ Department of Hepatobiliary Surgery, The Sixth People’s Hospital of Chengdu, Chengdu, Sichuan, China; ^3^ Department of Radiology, The First People’s Hospital of Neijiang, Neijiang, Sichuan, China; ^4^ Department of Pathology, The First People’s Hospital of Neijiang, Neijiang, Sichuan, China; ^5^ Department of Oncology, The First People’s Hospital of Neijiang, Neijiang, Sichuan, China

**Keywords:** sarcomatoid hepatocellular carcinoma, TKI, anti-PD-1 antibody, liver tumor, targeted-immunotherapy

## Abstract

Sarcomatoid hepatocellular carcinoma (SHC) is an aggressive malignancy with poor therapeutic outcomes. Current evidence supports radical surgical resection as the primary treatment modality, yet it is associated with prohibitively high postoperative recurrence rates. We report the clinical course of a 66-year-old male diagnosed with SHC. A hepatic mass (4.1 × 2.7 × 4.4 cm) was incidentally detected during routine health screening. The patient underwent laparoscopic right posterior sectionectomy, with histopathological confirmation of SHC. Superior mesenteric lymphadenopathy suspicious for metastasis developed 1 month postoperatively. Prophylactic transarterial chemoembolization was initiated, followed by 6-month tyrosine kinase inhibitor (TKI) therapy, achieving disease stabilization. However, lymph nodes progression occurred after TKI discontinuation, confirming metastatic involvement. Subsequent combined therapy (a TKI and an anti-PD-1 antibody) was administered. Notably, the patient self-discontinued treatment after two cycles, yet subsequent imaging revealed complete resolution of metastatic lymphadenopathy. No additional antitumor therapy has been administered, and the patient remains recurrence-free with 18-month overall survival confirmed by recent radiographic surveillance.

## Introduction

Sarcomatoid hepatocellular carcinoma (SHC) is a rare subtype of hepatocellular carcinoma (HCC), accounting for approximately 0.09% to 3.9% of all cases (1–3). The pathogenesis of SHC remains unclear, and its prognosis is extremely poor due to the high risk of recurrence and metastasis, coupled with the lack of effective pharmacological treatments. This report describes a case of SHC in which the patient developed superior mesenteric lymph nodes (SMLNs) metastasis after surgical resection. Following treatment with transarterial chemoembolization (TACE), tyrosine kinase inhibitors (TKI), and a short-term combination therapy (TKI and anti-programmed cell death [PD]-1 antibody), the SMLNs resolved. The patient has currently survived for 18 months with no evident signs of tumor recurrence.

## Case presentation

### Diagnosis and treatment

**Figure 1:**
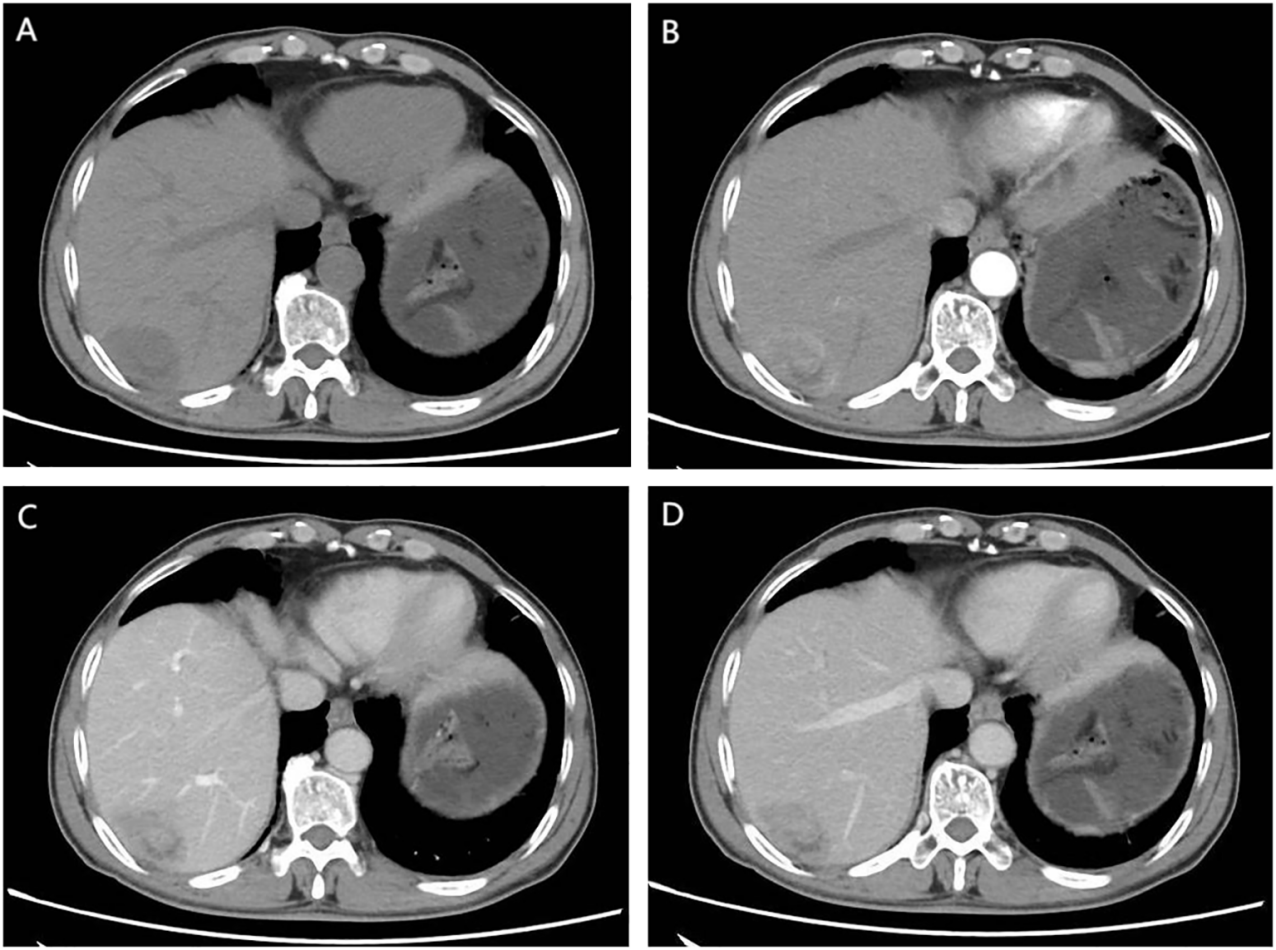
CT Findings: **(A)** Unenhanced CT scan. An irregular soft tissue mass in the subcapsular region of the right posterior hepatic lobe shows heterogeneous density without capsular retraction or biliary dilation. **(B)** Early arterial phase. Thickened arteries are seen surrounding the lesion. The mass exhibits marked heterogeneous enhancement with internal non-enhancing areas. **(C)** Portal venous phase. The lesion remains heterogeneously enhanced. The peripheral portion shows mild washout compared to the prior phase, while nodular enhancement emerges in the central region. **(D)** Delayed phase. Further peripheral washout is observed. Central nodular enhancement persists with slight decrease in intensity.

A 66-year-old male was admitted to the hospital due to the discovery of a liver tumor. One week prior to admission, the patient underwent an abdominal ultrasound during a routine physical examination, which revealed a mass in the right posterior lobe of the liver. Further contrast-enhanced CT of the upper abdomen indicated imaging features consistent with a malignant liver tumor, measuring 4.1 cm × 2.7 cm × 4.4 cm (**Figure 1**). The patient had a history of hepatitis C, which had been cured with antiviral therapy two years before admission. He also had a history of diabetes mellitus, currently managed with regular oral acarbose and metformin, resulting in well-controlled blood glucose levels. The patient denied other medical or family histories. Physical examination revealed no significant positive findings. Laboratory tests showed a positive hepatitis C antibody (+), while other results including complete blood count (WBC: 5.31x10 (9)/L, NEUT%: 67.5%), liver function tests (TBIL: 14.5 umol/L, ALT: 38 U/L, AST: 25 U/L, ALB:41.8 g/L), coagulation profile, renal function tests, alpha-fetoprotein (AFP: 2.92 ng/ml), protein induced by vitamin K absence or antagonist-II (PIVKA-II: 16.1 mAU/ml), and Carbohydrate Antigen 19-9 (CA19-9: 18 IU/mL) were all within normal ranges. Subsequent contrast-enhanced MRI further confirmed the presence of a malignant liver lesion, suggestive of primary HCC (**Supplementary Figure 1**). The patient was classified as Child-Pugh A for liver function and had an Eastern Cooperative Oncology Group (ECOG) performance status score of 0.

**Figure 2:**
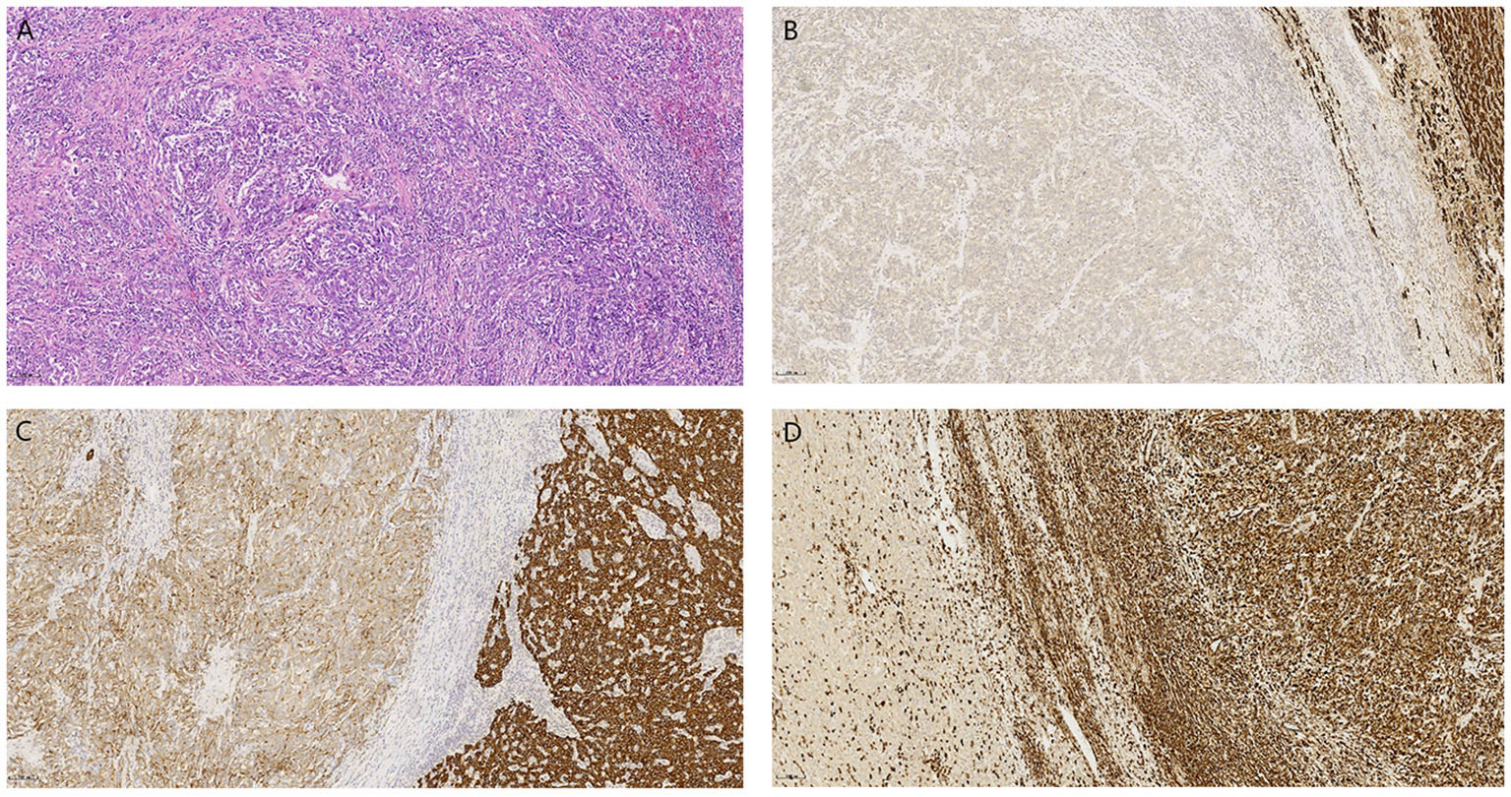
**(A)** (H&E, 200×) demonstrates disorganized short spindle-shaped tumor cells with residual normal hepatocytes in the upper right corner. Immunohistochemistry reveals tumor cells negative for HepPar-1 **(B)** and Vimentin **(D)**, with weak CK8/18 expression **(C)**, contrasting adjacent normal hepatocytes showing preserved positivity for these markers (IHC, 100×).

The patient underwent laparoscopic right posterior sectionectomy of the liver one week after admission, followed by pathological examination. Integrated with morphological and immunohistochemical findings, the pathological diagnosis was consistent with hepatocellular carcinoma with extensive sarcomatoid transformation (70% sarcomatoid component). Key features included: Edmondson-Steiner grade IV differentiation; histologic subtypes comprising macrotrabecular-massive and solid patterns with spindle cell sarcomatoid morphology; prominent stromal lymphocytic infiltration and fibrosis; evident tumor necrosis; presence of vascular and microvascular invasion; no definite perineural invasion or satellite nodules; and negative surgical margins. Immunohistochemical analysis revealed a biphasic immunophenotype. The conventional HCC component exhibited positivity for HepPar-1, CK8/18, and focal CK7, while demonstrating loss of AE1/AE3. The sarcomatoid component showed weak and focal expression of AE1/AE3, CK8/18, and CK19, strong diffuse vimentin positivity, and complete absence of hepatocytic markers (HepPar-1). CD34 highlighted microvascular density. The high proliferative index (Ki-67 ≈80%) correlated with the aggressive histology. Negative staining for Melan-A, HMB45, S-100, Syn, CD117, CD10, and Desmin excluded melanoma, neuroendocrine, and mesenchymal differentials. This immunophenotypic profile conclusively supports SHC with bidirectional differentiation, epithelial-mesenchymal transition, and high-grade malignancy. (**Figure 2**). The postoperative recovery was uneventful, and the patient was discharged on postoperative day 8.

### Postoperative follow-up and treatment

**Figure 3:**
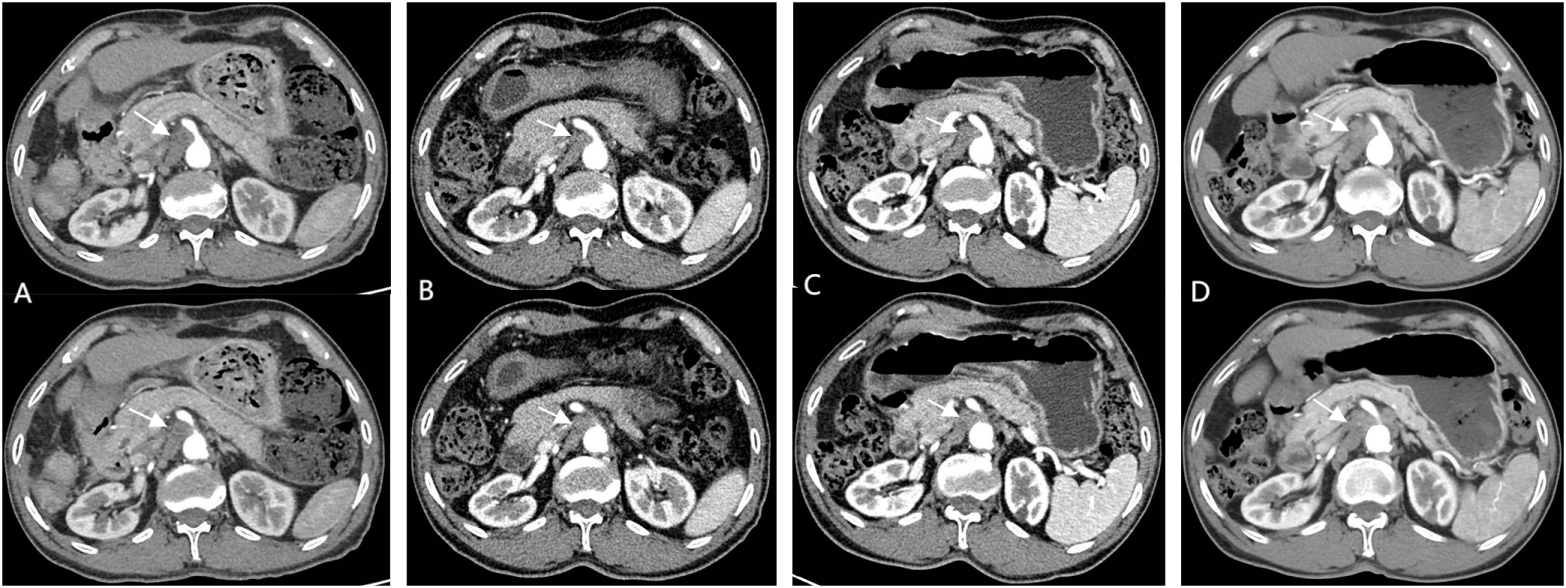
**(A)** (1.5 months post-operation) shows significant enlargement of SMLNs; **(B, C)** (2/4 months after Lenvatinib initiation) demonstrate treatment response; **(D)** (1-month post-Lenvatinib discontinuation) reveals significant re-enlargement SMLNs.

Given the patient’s high-risk factors for recurrence, prophylactic transarterial chemoembolization (TACE) was recommended to be performed approximately one month postoperatively after physical recovery. However, this recommendation was extrapolated from treatment guidelines established for conventional HCC rather than evidence specific to SHC (4, 5). The patient subsequently returned to the outpatient clinic 40 days after discharge, for re-examination. Imaging revealed progressive enlargement of SMLNs compared to preoperative findings, raising suspicion of lymph node metastasis (**Figure 3A**). Due to the presence of high recurrence risk and enlarged mesenteric lymph nodes, coupled with the absence of definitive treatment guidelines, a multidisciplinary team discussion was conducted. After thorough communication with the patient, the original prophylactic TACE regimen was maintained, with the addition of targeted therapy. The patient underwent prophylactic TACE and initiated oral lenvatinib mesilate (8mg once daily) upon hepatic function recovery. Regular outpatient follow-ups with tri-monthly imaging surveillance were arranged. Subsequent follow-ups demonstrated no new lesions. Notably, the mesenteric lymph nodes showed initial size reduction at the first re-evaluation and remained stable thereafter (**Figure 3B, C**). After six months of disease stability without conclusive radiological evidence of metastasis, lenvatinib was discontinued, while outpatient monitoring continued.

A repeat contrast-enhanced CT scan performed one-month after discontinuation of lenvatinib revealed no new lesions elsewhere but demonstrated significant re-enlargement of mesenteric lymph nodes. Combined with clinical history, this confirmed lymph node metastasis (**Figure 3D**). Combination targeted-immunotherapy (TKI and anti-PD-1 antibodies) was advised. The patient initiated the first cycle (Bevacizumab 750mg IV + Sintilimab 200mg IV administered in 21-day cycles) one month after follow-up. However, after two cycles, treatment was subsequently self-discontinued due to financial constraints, though outpatient surveillance continues without further pharmacological intervention.

### Subsequent follow-up

**Figure 4:**
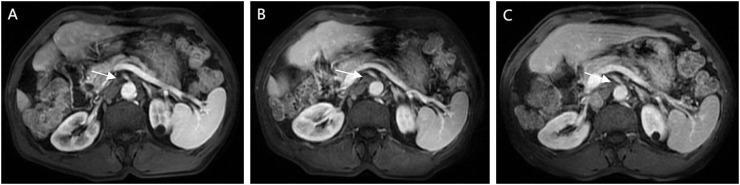
Follow-up CEMRI scans reveal complete resolution of previously enlarged lymph nodes, with images **(A)** (13 months post-operative), **(B)** (15 months post-operative), and **(C)** (18 months-postoperative) documenting temporal changes.

The patient returned for follow-up 45 days after discontinuation of the medication. A contrast-enhanced abdominal MRI revealed complete resolution of the previously enlarged SMLNs (**Figure 4A**). Subsequent tri-monthly follow-up evaluations demonstrated no recurrence of mesenteric lymphadenopathy (**Figure 4B**). The most recent surveillance conducted on February, 2025 (18 months since disease onset) showed no evidence of tumor recurrence (**Figure 4C**). The diagnostic and therapeutic timeline is summarized in **Supplementary Figure 2**.

## Discussion

SHC, a prognostically unfavorable subtype of HCC, exhibits aggressive clinicopathological features including high metastatic potential and frequent recurrence. Its low incidence has resulted in limited large-scale studies. Sih-Han Liao et al. (6) reported 1-, 3-, and 5-year overall survival (OS) rates of 45.0%, 17.5%, and 10.0% in 40 SHC patients, with 6-month and 2-year recurrence rates of 30.0% and 70.0% among 20 patients undergoing curative resection. Lu Wu et al. (3) analyzed 147 SHC cases from the National Cancer Database (NCDB), demonstrating even poorer survival outcomes (1-, 3-, and 5-year OS: 20.4%, 8.0%, 5.7%), though AJCC Stage I patients showed significantly better OS compared to advanced-stage counterparts. Recent data from Rong-Qi Sun et al. (7) involving 46 SHC patients revealed 1-, 3-, and 5-year OS rates of 61.3%, 28.0%, and 16.8% following curative surgery, with corresponding recurrence-free survival (RFS) rates declining from 40.0% to 0.0% over the same period. These collective findings underscore the historically dismal therapeutic efficacy in SHC management.

Previous studies (6, 8) demonstrate significantly higher lymph node metastasis rates in SHC compared to conventional HCC, though the underlying mechanisms remain elusive. Our case aligns with this pattern: despite a small tumor size and curative resection, markedly enlarged SMLNs were detected within one month postoperatively. Metastatic involvement was inferred based on the SHC diagnosis and absence of other malignancies on comprehensive imaging. Retrospective review of preoperative CT, however, revealed preexisting suspicious superior mesenteric lymphadenopathy—indicating metastasis likely occurred preoperatively. This finding was initially underappreciated due to the low incidence of nodal metastasis in conventional HCC.

Regarding the imaging manifestations of SHC, a study by He et al. (9) on dynamic contrast-enhanced CT of 13 patients with sarcomatoid HCC demonstrated that the primary lesion was more frequently located in the right lobe (84.6%, 11/13) than in the left lobe. The masses were mostly irregular in shape, with 7 cases showing central ischemic necrosis. Additionally, dynamic enhanced CT in 8 patients revealed a “slow-in and slow-out” pattern, characterized by peak enhancement occurring during the portal venous phase. Similarly, Liao et al. (6) observed central necrosis with peripheral arterial enhancement in ~50% of 40 SHC cases, while only 47.5% showed typical HCC enhancement kinetics. Koo et al. (10) corroborated these atypical imaging findings. The CT findings in our patient closely resembled prior studies but with slight differences, and demonstrated notable distinctions from the imaging manifestations of conventional HCC. Notably, the tumor exhibited significant central necrosis along with intratumoral enhancement during the arterial phase. Crucially, persistent enhancement was observed in the central region of the tumor during both the portal venous and delayed phases, presenting an early enhancement with late washout pattern (early-in and late-out), without the complete peripheral washout characteristic of conventional HCC. This atypical enhancement pattern may reflect the rapid growth biology of SHC (9).

Emerging evidence offers cautious optimism. Recent reports (2, 11–13) describe advanced SHC cases achieving favorable responses through multimodal strategies (e.g., TKIs, immunotherapy, and surgery), providing valuable insights for clinical decision-making. Furthermore, a case report by Liang et al. (2) describing a 69-year-old male with SHC demonstrated tumor reduction following combined targeted and immunotherapy, which subsequently enabled curative resection. The patient received postoperative combination therapy for 6 months and showed no signs of recurrence at the time of publication. This case suggests potential benefit from both preoperative and postoperative adjuvant therapy in such patients. Remarkably, our patient achieved an unexpectedly favorable therapeutic outcome: targeted therapy halted disease progression, and subsequent two cycles of combined therapy achieved complete radiologic resolution of SMLNs. This outcome is particularly notable given the abbreviated treatment duration due to financial constraints. Regrettably, the rarity of SHC has limited the available evidence for its systemic treatment, with current reports of effective pharmacological interventions primarily relying on empirical approaches and isolated case studies. Encouragingly, research on immunohistochemical and genomic profiling of SHC is rapidly expanding. Multiple studies (7, 14–16) are elucidating the evolutionary trajectory of SHC, providing a rationale for future targeted therapeutic strategies. Furthermore, consistent reports (17–19) of PD-L1 overexpression in SHC suggest potential benefit from immunotherapy in this patient population. It is hoped that further investigations will translate these findings into improved clinical outcomes for SHC patients.

Our management of this case highlights several limitations and areas for further discussion. First, the diagnosis of SMLN metastasis relied solely on imaging findings without histopathological confirmation via biopsy or metabolic verification through PET-CT. Second, retrospective review of preoperative contrast-enhanced CT images revealed preexisting suspicious enlargement of SMLNs, indicating suboptimal preoperative staging that may have impacted clinical decision-making. Lastly, although prophylactic TACE was administered postoperatively, we cannot provide definitive evidence regarding its efficacy in preventing recurrence in SHC.

## Conclusion

In conclusion, SHC represents a highly aggressive malignant tumor with poor prognosis. Current research findings consistently demonstrate that radical surgical resection remains the primary treatment option, yet the overall survival outcomes remain suboptimal. Notably, tyrosine kinase inhibitors (TKIs) and anti-PD-1 antibodies have exhibited significant therapeutic benefits in selected case reports, including the case study we reported. These preliminary findings warrant further investigation through rigorously designed clinical trials and may provide guidance for future therapeutic decision-making. However, large-scale multicenter studies with robust methodological quality are still required to comprehensively evaluate the efficacy of various treatment modalities and establish evidence-based clinical guidelines.

## Data Availability

The original contributions presented in the study are included in the article/**Supplementary Material**. Further inquiries can be directed to the corresponding author.

## Supplementary material

Supplementary Figure 1 MRI Findings: A soft tissue mass in the right posterior liver lobe demonstrates high signal on T2WI (A), low signal on T1WI (B/C), and restricted diffusion with high DWI signal and reduced ADC at the lesion periphery. Internally, it contains patchy T2-hyperintense areas with relatively regular morphology and well-defined margins, without capsular retraction or biliary dilation (D/E). Post-contrast images show arterial phase hyperenhancement with non-enhancing areas, venous phase peripheral washout with internal nodular enhancement, and persistent nodular enhancement during the delayed phase, reflecting “fast-in and slow-out” kinetics at the periphery and progressive enhancement internally (F–H).

Supplementary Figure 2 Timeline of the patient’s clinical course.
