# Supplementary figures and images for: Curative response to combined targeted-immunotherapy for post-hepatectomy lymph node metastasis in sarcomatoid hepatocellular carcinoma: case report and literature review
Source: Front Oncol. 2025 Sep 18;15:1591419. doi: 10.3389/fonc.2025.1591419 (PMC12488422; doi:10.3389/fonc.2025.1591419)

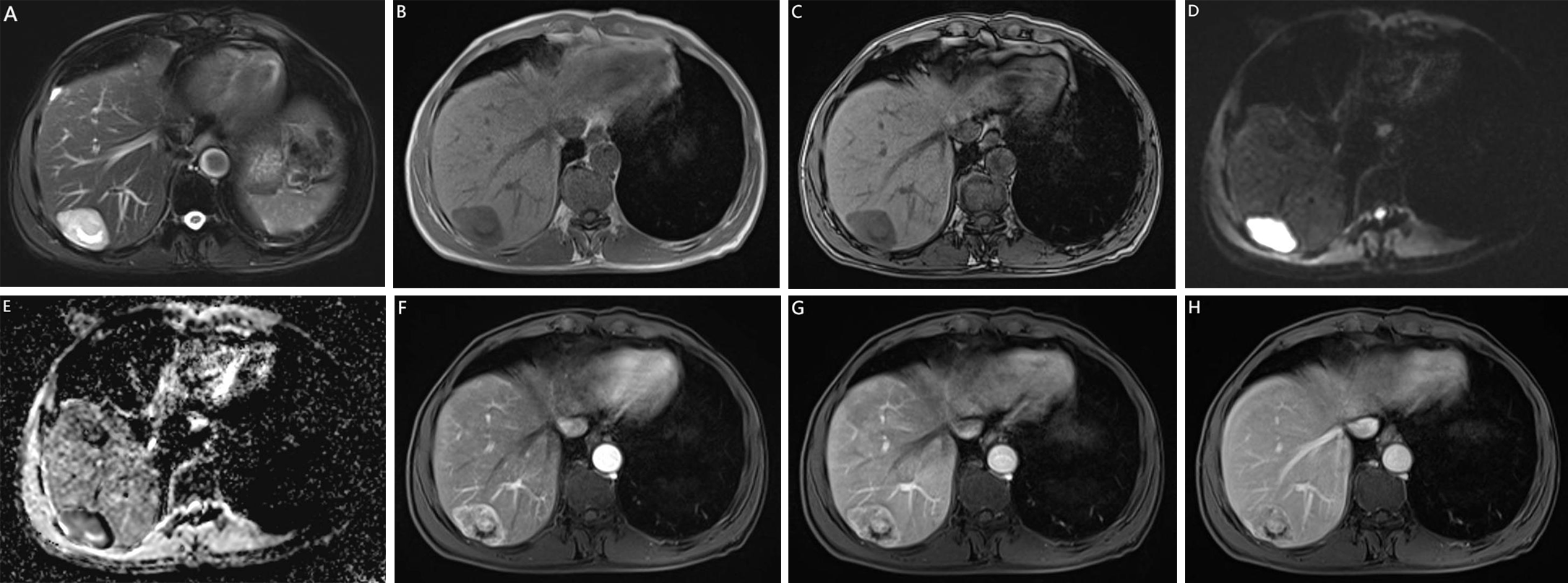

Supplement: Supplementary Figure 1 — MRI Findings: A soft tissue mass in the right posterior liver lobe demonstrates high signal on T2WI (A), low signal on T1WI (B/C), and restricted diffusion with high DWI signal and reduced ADC at the lesion periphery. Internally, it contains patchy T2-hyperintense areas with relatively regular morphology and well-defined margins, without capsular retraction or biliary dilation (D/E). Post-contrast images show arterial phase hyperenhancement with non-enhancing areas, venous phase peripheral washout with internal nodular enhancement, and persistent nodular enhancement during the delayed phase, reflecting “fast-in and slow-out” kinetics at the periphery and progressive enhancement internally (F–H). [file Image1.tif]

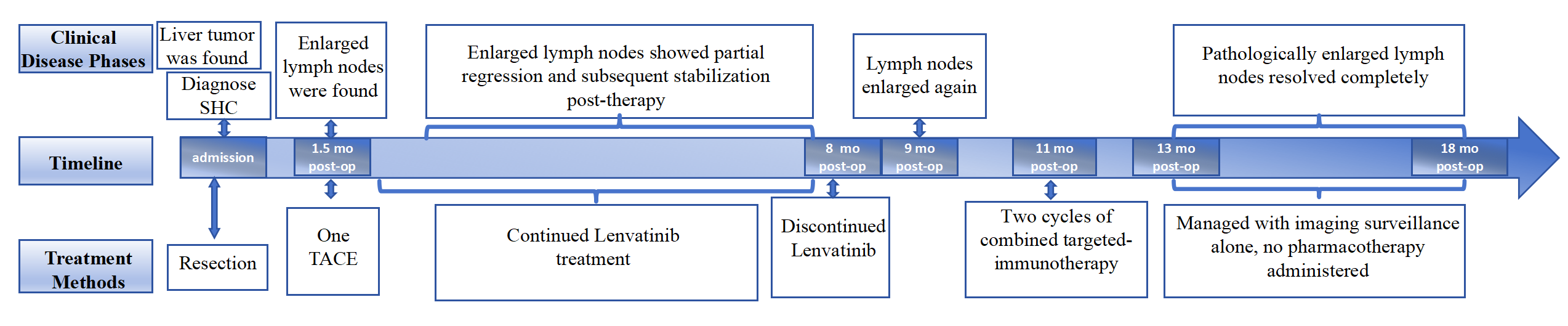

Supplement: Supplementary Figure 2 — Timeline of the patient’s clinical course. [file Image2.tif]
